# Stabilized homoserine o-succinyltransferases (MetA) or L-methionine partially recovers the growth defect in *Escherichia coli* lacking ATP-dependent proteases or the DnaK chaperone

**DOI:** 10.1186/1471-2180-13-179

**Published:** 2013-07-30

**Authors:** Elena A Mordukhova, Dooil Kim, Jae-Gu Pan

**Affiliations:** 1 Superbacteria Research Center, Korea Research Institute of Bioscience and Biotechnology (KRIBB), 111 Gwahangno, Yuseong-gu, Daejeon 305-806, South Korea

**Keywords:** *Escherichia coli*, Thermostability, Homoserine o-succinyltransferase (MetA), Growth rate, ATP-dependent proteases, DnaK chaperone

## Abstract

**Background:**

The growth of *Escherichia coli* at elevated temperatures is limited due to the inherent instability of homoserine *o*-succinyltransferase, MetA, which is the first enzyme in the methionine biosynthesis pathway. MetA is also unstable under other stressful conditions, such as weak organic acids and oxidative stress. The MetA protein unfolds, even at 25°C, forms considerable aggregates at 37°C and completely aggregates at 44°C.

**Results:**

We extended the MetA mutation studies using a consensus concept based on statistics and sequence database analysis to predict the point mutations resulting in increased MetA stability. In this study, four single amino acid substitutions (Q96K, I124L, I229Y and F247Y) in MetA designed according to the consensus concept and using the I-mutant2.0 modeling tool conferred accelerated growth on the *E. coli* strain WE at 44°C*.* MetA mutants that enabled *E. coli* growth at higher temperatures did not display increased melting temperatures (T_*m*_) or enhanced catalytic activity but did show improved *in vivo* stability at mild (37°C) and elevated (44°C) temperatures. Notably, we observed that the stabilized MetA mutants partially recovered the growth defects of *E. coli* mutants in which ATP-dependent proteases or the DnaK chaperone was deleted. These results suggest that the impaired growth of these *E. coli* mutants primarily reflect the inherent instability of MetA and, thus, the methionine supply. As further evidence, the addition of methionine recovered most of the growth defects in mutants lacking either ATP-dependent proteases or the DnaK chaperone.

**Conclusions:**

A collection of stable single-residue mutated MetA enzymes were constructed and investigated as background for engineering the stabilized mutants. In summary, the mutations in a single gene, *metA,* reframe the window of growth temperature in both normal and mutant *E. coli* strains*.*

## Background

Methionine is an essential amino acid in mammalian cells, although most bacteria, fungi and plants synthesize this amino acid *de novo* from aspartate [1]. Methionine participates in protein biosynthesis both as an initial amino acid and as one of the basic building blocks [2]. In *Escherichia coli,* the first enzyme in the methionine biosynthesis pathway, homoserine *o*-succinyltransferase (MetA) [1,3-5], is extremely sensitive to many stress conditions (e.g., thermal, oxidative or acidic stress) [6-8]. At temperatures higher than 25°C, MetA activity is reduced, and the protein tends to unfold, resulting in a methionine limitation in *E. coli* growth [9]. MetA reversibly unfolds at temperatures approaching 42°C and is a substrate for the ATP-dependent proteases Lon, ClpP/X and HslVU [6]. At temperatures of 44°C and higher, MetA completely aggregates and is no longer found in the soluble protein fraction, thus limiting growth [9]. The chemical chaperone trimethylamine oxide reduces insoluble MetA accumulation and improves *E. coli* growth at elevated temperatures [9]. It has been suggested that MetA could be classified as a Class III substrate for chaperones because this molecule is extremely prone to aggregation [10].

Despite the importance of MetA in *E. coli* growth, little information exists on the amino acid residues involved in the inherent instability of MetA. The sensitivity of MetA to multiple stress conditions suggests that this enzyme might be a type of ‘metabolic fuse’ for the detection of unfavorable growth conditions [7]. Previously, we used random mutagenesis of *metA* to improve *E. coli* growth at elevated temperatures [11]. Mutations that resulted in the amino acid substitutions I229T and N267D enabled the *E. coli* strain WE to grow at higher temperatures and increased the ability of the strain to tolerate acidic conditions. In this study, we extended our stabilization studies using a computer-based design and consensus approach [12] to identify additional mutations that might stabilize the inherently unstable MetA enzyme. To achieve pronounced thermal stabilization, we combined several single substitutions in a multiple mutant, as the thermo-stabilization effects of individual mutations in many cases were independent and nearly additive [12]. Here, we describe the successful application of the consensus concept approach and the I-mutant2.0 modeling tool [13] to design stabilized MetA mutants. The consensus concept approach for engineering thermally stable proteins is based on an idea that by multiple sequence alignment of the homologous counterparts from mesophiles and thermophiles, the nonconsensus amino acid might be determined and substituted with the respective consensus amino acid, contributing to the protein stability [12]. I-Mutant2.0 is a support vector machine-based web server for the automatic prediction of protein stability changes with single-site mutations (http://gpcr.biocomp.unibo.it/~emidio/I-Mutant2.0/I-Mutant2.0_Details.html).

Four substitutions, Q96K, I124L, I229Y and F247Y, improved the growth of the *E. coli* WE strain at elevated temperatures. Unexpectedly, the MetA mutants I124Y and I229Y, which conferred higher growth rates at 44°C, displayed melting temperatures similar to that of the native enzyme but exhibited improved *in vivo* stability. The stabilized MetA mutant enzymes at least partially recovered the growth defects of mutant *E. coli* strains with deletions of either ATP-dependent proteases or the DnaK chaperone. These results suggest that the growth defects of Δ*dnaK* or protease-deficient mutants primarily reflect malfunctioning MetA at 37°C, a standard physiological temperature. Consistently, the addition of methionine recovered the temperature-dependent growth defects of these mutants.

## Results

### Mutant MetAs enable *E. coli* growth at elevated temperatures

**Figure 1:**
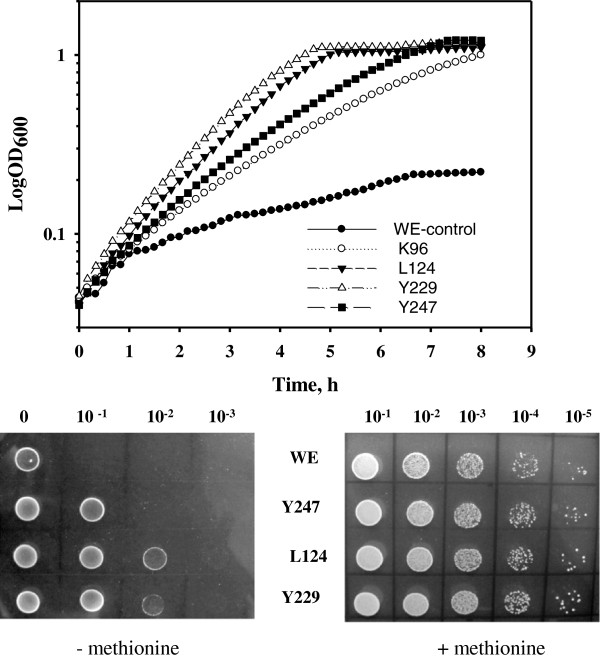
**Stabilized MetA mutants stimulate growth of the *E. coli *WE strain at 44°C.** The strains were cultured in M9 glucose medium in a TVS126MB automatic growth-measuring incubator at 44°C. The optical densities of the growing cultures were measured at 600 nm every 10 min. The average of two independent experiments is presented. Serial dilutions of cultures growing logarithmically at 30°C in M9 glucose medium (OD_600_ of 0.5) were spotted on M9 glucose and M9 glucose L-methionine (50 μg/ml) agar plates. The cells were incubated for 24 h at 44°C.

Previously, we identified two amino acid substitutions, I229T and N267D, which conferred stability to the MetA protein [11]. To obtain additional stable MetA mutants, we employed a multiple alignment approach and identified eight amino acid residues present in all thermophilic MetAs but absent in *E. coli* MetA (Additional file 1: Figure S1). The *metA* mutations that resulted in the corresponding amino acid substitutions Q96K, L110V, I124L, R160L, A195T, A200E, D218G and F247Y were integrated into the *E. coli* JW3973 (∆*metA*) chromosome to yield the strains K96, V110, L124, L160, T195, E200, G218 and Y247, respectively. Among the constructed strains, three mutants, K96, L124 and Y247, demonstrated accelerated growth at 44°C in M9 glucose medium (Figure 1; Additional file 2: Table S1) compared with the control strain WE, which harbored the wild-type *metA* gene from the *E. coli* K-12 strain W3110 [11].

Using the I-Mutant2.0 modeling tool [13] for protein stability prediction, the I229Y mutation was predicted to improve MetA stability and accelerate growth at 44°C (Figure 1; Additional file 2: Table S1). To confirm the enhanced thermo-tolerant growth of the L124, Y229 and Y247 mutants, the serially diluted cultures were incubated on solid M9 glucose plates at 44°C (Figure 1). The viability of the mutant strains was increased by at least one to two orders of magnitude compared with the wild-type strain (Figure 1). Supplementation of the culture medium with L-methionine stimulated the growth of the wild-type and the mutant strains at 44°C to the same extent, thus abolishing the differences between the wild-type and mutant strains (Figure 1). The mutant strains L124 and Y229, which displayed the higher growth rates at 44°C (Additional file 2: Table S1), were selected for further analysis.

To test the combinatorial effects of the mutations, we constructed mutant strains harboring I124L-I229Y and I124L-I229Y-N267D substitutions in the MetA enzyme (designated as LY and LYD, respectively). The N267D substitution conferring an increased thermal stability to the MetA enzyme has been previously described [11]. The double LY and triple LYD mutant strains were cultured at 45°C in M9 glucose medium and compared with single mutants L124 and Y229 and the wild-type strain WE (Additional file 3: Figure S2). The temperature 45°C was chosen because no significant differences between the strains harboring single and multiple mutated MetA enzymes were detected at 44°C (data not shown). The wild-type strain did not grow at 45°C (Additional file 3: Figure S2). The double LY and triple LYD mutants grew faster than the single mutant strains L124 and Y229, which had specific growth rates of 0.37 and 0.42 h^-1^ versus 0.18 and 0.3 h^-1^, respectively. The highest growth rate at 45°C was observed in the LYD strain (0.42 h^-1^), in which the effects of the MetA enzyme were combined the maximal number of the stabilizing mutations. However, the mutant LYD still grew slower than in the presence of L-methionine (specific growth rate 0.53 h^-1^; data not shown). This result might reflect the presence of another thermolabile protein in the methionine biosynthetic pathway. Previously, Mogk *et al.*[14] showed that MetE, which catalyzes the last step in methionine biosynthesis, was also thermally sensitive and tended to form aggregates at a 45°C heat shock.

### Mutant MetAs enabling *E. coli* growth at higher temperatures did not display an increased thermal transition midpoint

To determine whether the accelerated growth observed at 44°C for the single mutant MetA strains is due to increased thermal stability of MetA, the protein melting temperature (*T*_m_) was measured using differential scanning calorimetry (DSC). The wild-type and mutant MetA enzymes containing a C-terminal six-histidine tag were purified as described in the Methods section. The *T*_m_ of the wild-type MetA was 47.07 ± 0.01°C (Table 1), and the *T*_m_s of the stabilized MetA proteins were slightly higher than that of the wild-type enzyme (Table 1).

**Table 1:** Differential scanning calorimetric data for the wild- type and mutant MetA enzymes

**Enzyme**	***T***_**m **_**(**°**C)**	∆H^*^	**∆H**_v_^*^	**∆H/∆H**_**v**_
MetA, wt	47.01 ± 0.26	5.93 x 10^4^	1.18 x 10^5^	0.5
I124L	48.65 ± 0.06	6.51 x 10^4^	1.86 x 10^5^	0.35
I229Y	50.68 ± 0.06	8.99 x 10^4^	2.38 x 10^5^	0.38

^*^The errors associated with the data were <2% for ∆H and ∆H_v_. The calorimetric heat (∆H) is the heat change per mole of enzyme. The van’t Hoff heat (∆H_v_) is the heat change per cooperative unit. The ratio ∆H/∆H_v_ is a measure of the number of thermally transited cooperative units per mole of enzyme. All measurements were performed in triplicate.

Because the stabilized mutants displayed *T*_m_ values similar to the native enzyme, we hypothesized that the catalytic activity was enhanced in the MetA mutants. No difference was observed in the *k*_cat_ and *K*m values for succinyl-CoA between the stabilized MetA mutants and native MetA enzyme, whereas the *K*m for L-homoserine was reduced 1.5-fold in the I124L mutant compared with the wild-type MetA (Table 2). This finding is consistent with the slight increase in *k*_cat_/*K*m of 58% compared with the native enzyme. Thus, the stabilizing mutations had little to no effect on the catalytic activity of the MetA enzyme.

**Table 2:** Kinetic parameters of the wild-type and stabilized MetA enzymes

**Enzyme**	***k***_**cat **_**(s**^**-1**^**)**	**Succinyl-CoA**		**L-homoserine**	
		***K***_**m **_**(mM)**	***k***_**cat**_**/*K***_**M **_**(M**^**-1**^ **s**^**-1**^**)**	***K***_**m **_**(mM)**	***k***_**cat**_**/*K***_**M **_**(M**^**-1**^ **s**^**-1**^**)**
MetA, wt	36.72 ± 0.9	0.37 ± 0.05	9.9*10^4^	1.25 ± 0.3	2.93*10^4^
I124L	38.59 ± 0.5	0.38 ± 0.06	1.02*10^5^	0.83 ± 0.15	4.65*10^4^
I229Y	39.28 ± 0.5	0.36 ± 0.06	1.09*10^5^	1.42 ± 0.1	2.76*10^4^

### MetA mutant enzymes exhibit reduced aggregation at an elevated temperature (45°C) *in vitro* and *in vivo*

**Figure 2:**
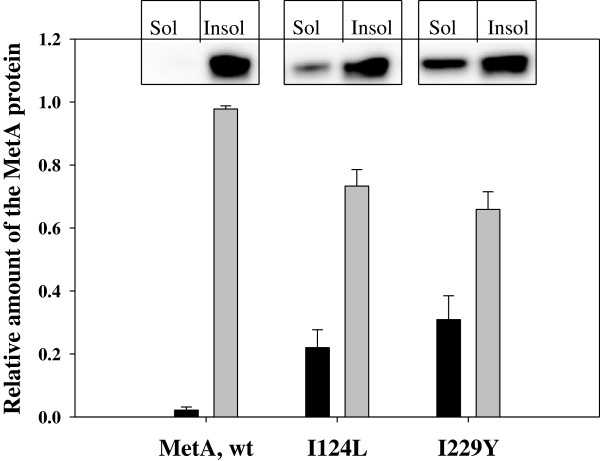
**Heat-induced aggregation of native and mutant MetAs *in vitro*.** Aggregated proteins were prepared through incubation at 45°C for 30 min as described in the Methods section; the soluble (black columns) and insoluble (gray columns) protein fractions were separated by centrifugation at 14,000 g for 30 min and analyzed through Western blotting with rabbit anti-MetA antibodies. The densitometric analysis of band intensity was conducted using WCIF Image J software. The total amount of MetAs before an incubation was equal to 1. The error bars represent the standard deviations of duplicate independent cultures.

Native MetA was previously reported to become completely aggregated *in vitro* at temperatures of 44°C and higher [9]. To examine the aggregation-prone behavior of native and stabilized MetAs, we generated *in vitro* aggregates of the purified proteins as described in the Methods section. The native MetA enzyme was completely aggregated after heating at 45°C for 30 min (Figure 2). In contrast, the engineered I124L and I229Y mutant MetAs demonstrated a higher level of aggregation resistance; only 73% of I124L and 66% of I229Y were insoluble (Figure 2).

In addition, we examined the level of soluble MetA enzymes *in vivo* after heat shock at 45°C for 30 min (Additional file 4: Figure S3). The amount of the native MetA protein in the soluble fraction decreased to 52% following heat shock, whereas the relative amounts of soluble MetA I124L and I229Y mutants were 76% and 68%, respectively. The amount of insoluble native MetA protein increased 28-fold after heating, while those of stabilized MetA I124L and I229Y mutants increased 20- and 17-fold, respectively (Additional file 4: Figure S3). These results confirmed the higher resistance of the stabilized I124L and I229Y mutant enzymes to aggregation.

### MetA mutant enzymes are more stable *in vivo* at normal (37°C) and elevated (44°C) temperatures

**Figure 3:**
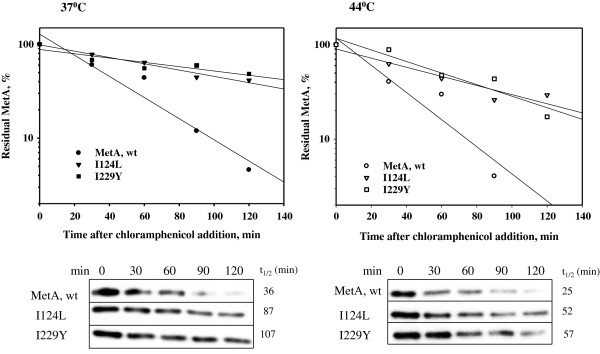
***In vivo *stability of MetA mutants.** Cells of the strains WE, L124 and Y229 exponentially growing (OD_600_ = 0.3) at 37°C in M9 medium were treated with 200 μg/ml of chloramphenicol. The cultures were divided; one half of each culture was maintained at 37°C (solid symbols), and the other half of the culture was shifted to 44°C (open symbols). The samples were collected at the indicated time points and analyzed through Western blotting as described in the Methods section. Densitometry results were normalized after setting the MetA amount before chloramphenicol addition equal to 100%.

To determine the effects of these mutations on MetA stability *in vivo*, we analyzed the degradation of the mutant and native MetA enzymes after blocking protein synthesis using chloramphenicol. The residual MetA in the cells was quantified through Western blotting as described in the Methods section. As shown in Figure 3, the I124L and I229L MetA mutants were approximately 2-3-fold more stable than native MetA, with half-lives (t_1/2_) of 87 min (I124L) and 107 min (I229L) at 37°C and 52 min (I124L) and 57 min (I229L) at 44°C, respectively; the half-life of the native MetA was 36 min at 37°C and 25 min at 44°C.

### Stabilized MetAs partially compensate the growth defects of the Δ*dnaK* mutants

**Figure 4:**
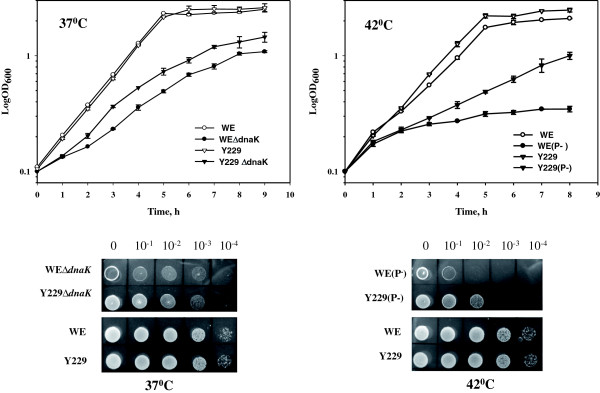
**Effect of stable MetA mutants on the growth of *dnaK *null and protease-deficient mutants of the *E. coli *strains WE and Y229.** The strains were cultured in 25 ml of M9 glucose medium in 125 ml Erlenmeyer flasks at 37°C (*∆dnaK* mutants) or 42°C (protease-minus mutants). To measure the growth, the optical density was monitored at 600 nm every 1 h. The average of two independent experiments is presented. Serial dilutions of logarithmically growing at 30°C (*∆dnaK* mutants) or 37°C (protease-minus mutants) in M9 glucose medium cultures (OD_600_ of 0.5) were spotted onto M9 glucose agar plates. The cells were incubated for 24 h at 37°C (*∆dnaK* mutants) or 42°C (protease-minus mutants).

MetA has been suggested to be classified as a Class III substrate for chaperones because this enzyme is extremely prone to aggregation [10]. Under physiological heat stress conditions, the DnaK system is the most effective chaperone for preventing the aggregation of thermolabile proteins [14]. Thus, the Δ*dnaK52* mutant strain displays a slower growth rate at 37°C and no growth at 42°C [15]. Because MetA is one of the most thermolabile proteins, we determined the growth profiles of *dnaK* null mutants expressing stabilized MetAs. We constructed the WE*∆dnaK*, L124*∆dnaK* and Y229*∆dnaK* mutant strains and cultured these cells in M9 glucose medium at 37°C. As shown in Figure 4, the mutant strain Y229*∆dnaK* grew 26% faster than the control strain WE*∆dnaK*, with a growth rate of 0.48 h^-1^ for Y229*∆dnaK* and 0.38 h^-1^ for WE*∆dnaK* (see Additional file 5: Table S2 for the specific growth rates). The mutant strain L124*∆dnaK* grew at the same rate as Y229*∆dnaK*. We observed an increased accumulation of insoluble wild-type MetA in heat-stressed *∆dnaK* cells compared with the mutated I124L and I229Y enzymes, which had relative amounts of 57% and 33% of the wild-type enzyme, respectively (Additional file 6: Figure S4). This finding might partially explain the slower growth of the WE*∆dnaK* strain due to an increased aggregation of the wild-type MetA compared with the I124L and I229Y mutants.

Despite an accelerated growth, the Y229*∆dnaK* mutant strain did not achieve the same growth rate as the *dnaK* + parental strain (Figure 4), potentially reflecting increased misfolding and the aggregation of other proteins in the absence the DnaK chaperone. We also examined the viability of serially diluted WE*∆dnaK* and Y229*∆dnaK* cultures at 37°C and confirmed the accelerated growth of the stabilized MetA mutant Y229*∆dnaK* (Figure 4). At 42°C, the non-permissive growth temperature for the *∆dnaK* mutants, no growth occurred, even in the presence of the stabilized MetA mutants (data not shown).

### Partial recovery of the impaired growth of protease-null mutants by the stabilized MetAs

Previous findings have revealed that the temperature-dependent unfolding of MetA resulted in the proteolysis of this enzyme [6]. Aggregated MetA is degraded by a combination of the ATP-dependent cytosolic proteases Lon, ClpPX/PA and HslVU, particularly at higher temperatures [6]. Because MetA is an inherently unstable protein, we reasoned that aggregated MetAs should be degraded by intracellular proteases and that protease-minus mutant, unable to degrade aggregated MetAs, would display hampered growth. The stabilized MetAs displaying higher *in vivo* stability would improve the growth of *E. coli* protease-negative mutants. The triple protease-deficient mutants WE(P^-^), L124(P^-^) and Y229(P^-^) were constructed and cultured at 42°C in M9 glucose-defined medium. Kanemori *et al.*[16] demonstrated the temperature-sensitive growth of the triple protease-deficient *E. coli* mutant KY2266 at 42°C. As shown in Figure 4, the mutant Y229(P^-^) exhibited an increased specific growth rate (μ) of 0.25 h^-1^ compared with a growth rate of 0.096 h^-1^ for the control strain WE(P^-^). The growth rate of L124(P^-^) was similar to that of Y229(P^-^) (Additional file 5: Table S3). These results indicate that the growth defect of the protease-deficient mutant might be a consequence of increased accumulation of the aggregated MetA proteins. Previously, Biran *et al.*[6] showed that the native MetA was stabilized in the cells of triple deletion mutant *lon, clpP, hslVU.* However, these authors did not identify which protein fraction, soluble or insoluble, contained the MetA. Apparently, an excess of the MetA synthesized at elevated temperatures in a proteolysis-minus background leads to the accumulation of insoluble aggregates that are toxic to the cells and inhibit bacterial growth. Therefore, we examined the *in vivo* aggregation of the wild-type and mutated MetA enzymes in heat-stressed protease-deficient cells. The relative amounts of MetA insoluble aggregates in the stabilized I124L and I229Y mutants were reduced to 59% and 44%, respectively, compared with wild-type MetA (Additional file 6: Figure S4). We assume that the stabilized MetAs remaining soluble and functionally active relieved the growth inhibition of the protease-negative *E. coli* mutant.

We also examine whether the stabilized MetAs affect the viability of protease-deficient strains at an elevated temperature (42°C). The mutant Y229(P^-^) was at least 10-fold more viable than the control strain WE(P^-^) (Figure 4). The same result was observed for the mutant L124(P^-^) (data not shown). However, despite accelerated growth and increased viability, the protease-deficient mutants harboring the stabilized MetAs grew slower than the protease-positive strains WE and Y229 (Figure 4). Our findings indicate that the growth defect in the protease-null mutant strain is partially due to MetA instability.

### Methionine recovers the growth defect of the *E. coli* mutants lacking either ATP-dependent proteases or the DnaK chaperone

**Figure 5:**
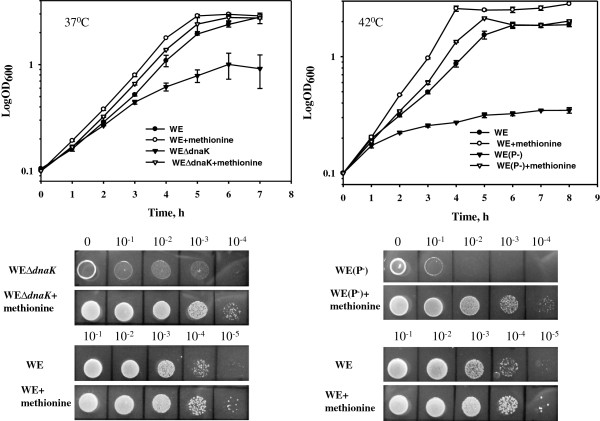
**L-methionine stimulates growth of Δ*dnaK *or protease-deficient mutants of the *E. coli *strain WE at non-permissive temperatures.** The strains were cultured in 25 ml of M9 glucose medium with or without L-methionine supplementation (50 μg/ml) in 125 ml Erlenmeyer flasks at 37°C (*∆dnaK* mutants) or 42°C (protease-minus mutants). The average of two independent experiments is presented. Serial dilutions of logarithmically growing at 30°C (*∆dnaK* mutants) or 37°C (protease-minus mutants) in M9 glucose medium cultures (OD_600_ of 0.5) were spotted onto M9 glucose or M9 glucose L-methionine (50 μg/ml) agar plates. The cells were incubated for 24 h at 37°C (*∆dnaK* mutants) or 42°C (protease-minus mutants).

Because the stabilized MetA mutants conferred an increased growth rate to *∆dnaK* and protease-deficient *E. coli* mutants at higher temperatures, we expected that methionine supplementation might recover the growth defects of both mutants. Thus, we examined the direct effect of L-methionine supplementation on WE*∆dnaK* and WE(P^-^) growth at 37°C and 42°C, respectively. In the methionine-supplemented medium, the mutants WE*∆dnaK* and WE(P^-^) grew two- and six-fold faster, respectively, than without L-methionine supplementation (Figure 5). For WE*∆dnaK*, the growth rate was 0.73 h^-1^ with methionine and 0.38 h^-1^ without methionine. For WE(P^-^), the growth rate was 0.58 h^-1^ with methionine and 0.095 h^-1^ without methionine (Figure 5; Additional file 5: Tables S2 and S3). The spot test confirmed the results obtained with flask-cultivation (Figure 5). L-methionine also stimulates the growth of the control strain WE at 37°C and 42°C (Figure 5; Additional file 5: Tables S2 and S3). However, the WE strain demonstrated only a 28% and 44% increase of the specific growth rates at 37°C and 42°C, respectively, in the presence of methionine (0.77 and 0.6 h^-1^ at 37°C; 0.78 and 0.54 h^-1^ at 42°C with and without methionine supplementation, respectively; Additional file 5: Tables S2 and S3). These results clearly indicate that an impaired methionine supply underlies the *dnaK*- and protease-null mutant growth defects.

To determine the effect of methionine on the growth of the mutated MetA strains, we cultivated the isogenic strains WE and WE*∆dnaK* and Y229 and Y229*∆dnaK* in the presence of methionine at 37°C (Additional file 7: Figure S5). In the methionine- supplemented medium, the *∆dnaK* mutants grew at equal rates, and only slightly slower growth than the *dnaK* + strains was observed (Additional file 5: Table S2; Additional file 7: Figure S5). These findings suggest that a malfunction of the methionine biosynthetic enzymes, including MetA, is primarily responsible for the impaired growth of the *∆dnaK* mutant strains at 37°C. At temperatures higher than 37°C, defects in other factors, such as chromosomal partitioning, extensive filamentation and increased levels of heat-shock protein (HSP) biosynthesis, might significantly hamper the growth of the Δ*dnaK* mutants, as previously shown for the Δ*dnaK52* mutant strain [15].

L-methionine also eliminated the difference in the growth rates between the protease- deficient control WE(P^-^) and mutant Y229(P^-^) strains (0.58 and 0.59 h^-1^, respectively) at 42°C (Additional file 5: Table S3; Additional file 7: Figure S5). However, the protease-negative mutants grew 25% slower than the parent strains in the presence of L-methionine (Additional file 5: Table S3; Additional file 7: Figure S5), potentially reflecting the accumulation of other protein aggregates [17].

A partial complementation of the impaired growth of the *∆dnaK* and protease-negative strains through stabilized MetAs indicates that the inherent instability of MetA plays a significant role in the growth defects observed in these mutant strains.

## Discussion

**Figure 6:**
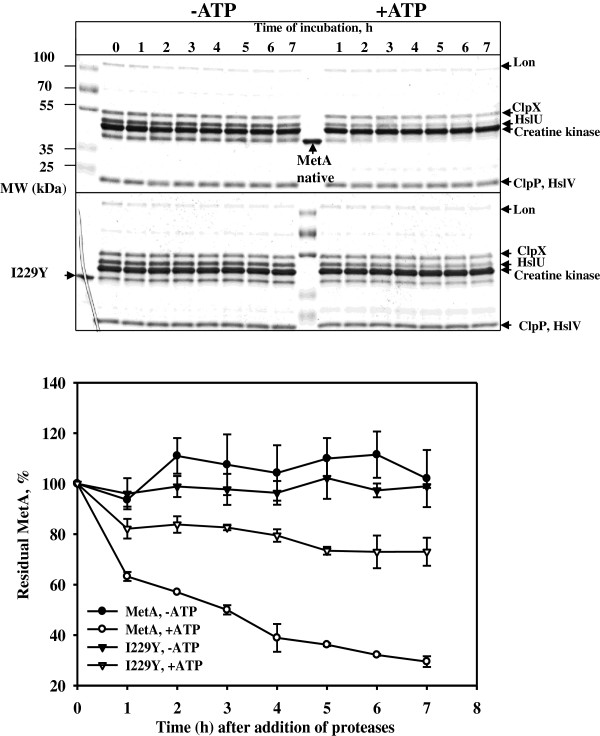
***In vitro *degradation of the native MetA protein and stabilized I229Y mutant by the ATP-dependent proteases Lon, ClpP/X and HslVU.** Degradation reactions were performed at 37°C with or without ATP as described in the Methods section. Untreated proteins indicate the positions of native MetA (the central lane of the upper gel) and mutant I229Y (the left lane of the lower gel). Densitometry results were normalized after setting the MetA amount before ATP addition equal to 100%. The results are plotted as the mean and standard deviation of two independent experiments.

The growth of *E. coli* strains at elevated temperatures in a defined medium is impaired by the extreme instability of the first enzyme in the methionine biosynthetic pathway, homoserine o-succinyltransferase (MetA) [18]. Although the key role of the MetA protein in *E. coli* growth under thermal stress has been known for 40 years [8], it is unclear which residues are involved in the inherent instability of MetA. Previously, we identified two amino acid substitutions, I229T and N267D, responsible for MetA tolerance to both thermal and acid stress [11]. In this study, we employed several approaches to design more stable MetA proteins*.* Using the consensus concept approach [12], stabilization was achieved through three single amino acid substitutions, Q96K, I124L and F247Y. We hypothesized that a combination of these amino acid substitutions might significantly increase MetA stability compared with the single mutants we identified in the randomly mutated thermotolerant MetA-333 [11]. The new MetA mutant enzymes were more resistant to heat-induced aggregation *in vitro* (Figure 2). The enhanced *in vivo* stabilities of the MetA mutants were also demonstrated through the immunodetection of residual MetA protein after blocking protein synthesis (Figure 3). However, the melting temperature, a good indicator of thermal stability [19], was only slightly increased. Instead of thermo-stabilization, the mutant MetAs might experience kinetic stabilization, in which a specific conformation change, which increases the unfolding barrier, ultimately results in slow unfolding rates [20]. This assumption is supported by a decreased level of the mutated MetAs observed in insoluble protein fraction under a temperature shift from 30° to 45°C compared with the native MetA protein (Additional file 4: Figure S3). If a native protein is thermodynamically unstable and/or functions under stress conditions, then kinetic stabilization could enhance the functional properties of the protein [21]. Furthermore, improved kinetic stability is tightly associated with protease resistance [22]. Notably, the MetA mutants were more resistant to proteases; *in vitro* reconstitution experiments confirmed the resistance of the MetA mutants to the ATP-dependent cytosolic proteases, including Lon, ClpPX/PA and HslVU (Figure 6). Previously, the aggregated MetA protein was identified as a substrate for intracellular proteases Lon, ClpPX/PA and HslVU [6]. Biran *et al.*[6] assumed the combinatorial action of these proteases on MetA degradation because the protein stabilization was detected in the triple deletion mutant *lon*, *clpP*, *hslVU* but not in any single (*lon*, *clpP*, *hflB* and *hslVU*) or double (*lon–clpP*) deletion mutants*.*

Previous studies have shown that the *dnaK* gene is not essential for growth and protein folding at 30°C but is required at temperatures above 37°C or below 15°C [23]. Here, we showed that the defective growth of a Δ*dnaK* mutant at 37°C can be partially restored using a stabilized MetA (Figure 4). This result suggests that the growth defect of the DnaK-deficient strain is primarily due to non-functional MetA because MetA, an inherently unstable protein even at the physiological temperature of 37°C, requires folding assistance from the DnaK chaperone system. The stabilized MetA mutants also partially restore the growth defects of protease-deficient strains at 42°C (Figure 4). We also examined whether the temperature-sensitive mutations (Δ*mukB*, Δ*bamE* and Δ*lpp*) affecting other cellular processes are suppressed through methionine supplementation at higher temperatures. None of the mutants showed improved growth, indicating that proper methionine supply is a major issue in the growth defects of both a *∆dnaK* and the triple protease mutants.

Taken together, these results suggest that the temperature-dependent growth defects of the Δ*dnaK* mutants and protease-deficient strains primarily reflect the malfunction of MetA and consequently, the methionine supply. Furthermore, the addition of methionine completely corrects the growth defect of the *dnaK* null mutant at 37°C and recovers most of the impaired growth of the protease-deficient strain at 42°C.

To evaluate the conformational changes caused by single-site mutations in the MetA protein, we performed molecular dynamics simulations of a homology model based on the closest MetA homolog, homoserine O-succinyltransferase from *Thermotoga maritima* (PDB code 2H2W). Our model has shown that the stabilizing MetA mutations were randomly distributed in different secondary structure elements (Additional file 8: Table S5). Stabilization has been shown for these mutants according to the altered free energy of protein folding (ΔΔG < −1 kcal/mol) (Additional file 8: Table S5). We observed that the highest ΔΔG value was correlated with the maximal melting temperature (T_*m*_) for the Y229 mutant (Table 1; Additional file 8: Table S5). We also calculated the cavity volume change as a parameter associated with the conformational stability and folding reaction [24]. The cavity volumes of all mutants were diminished compared with the native enzyme, with maximal decrease for the I229Y substitution (Additional file 8: Table S5). Cavities in proteins are a major contributor to low packing densities and reduced stability [25]. Cavities and surface grooves are also potential sites for the binding of drugs, ligands and other proteins [26]. Therefore, decreased cavity volumes should lead to higher conformational stability and resistance to aggregation for originally unstable proteins, such as MetA. Thus, MetA might be an inherently unstable protein [27] because it unfolds at room temperature and dramatically loses activity at 30°C or higher [9]. Due to its increased sensitivity to many stress conditions, including temperature, weak organic acids and oxidative stress [7], MetA protein has been suggested to function as a ‘metabolic fuse’ to detect unfavorable growth conditions [7].

## Conclusions

In this study, we further elucidated the mutations in MetA that facilitate faster *E. coli* growth at elevated temperatures (44°C) compared with the wild-type enzyme. Stabilized MetA proteins partially suppressed the temperature-sensitive phenotype of both *dnaK* and triple protease deficient mutants. Because improving the growth of *E. coli* at higher temperatures has an immediate application in realizing the bacterial cell factory, this improvement might also facilitate the identification of target genes and proteins, enabling thermotolerance or improved growth at higher operating temperatures [28-30].

## Methods

### Strains and culture conditions

The strains and plasmids used in this study are listed in Table 3.

**Table 3:** Bacterial strains and plasmids used in this study

**Strain or plasmid**	Relevant description^*^	**Source or reference**
*Escherichia coli*		
W3110	F-, *λ*^*-*^, *IN(rrnD-rrnE)1*, *rph-1*	KCTC
JW3973	F-, Δ*(araD-araB)567*, Δ*lacZ4787(::rrnB-3)*,	Keio collection
	*λ*^*-*^, rph-1, Δ*metA780::*kan,	National Institute
	Δ(*rhaD-rhaB)568*, *hsdR514,*	of Genetics, Japan
WE	JW3973 carrying the wild-type *metA* gene	[11]
K96	JW3973 carrying the *metA* gene	This study
	with the Q96K substitution	
L124	JW3973 carrying the *metA* gene	This study
	with the I124L substitution	
Y229	JW3973 carrying the *metA* gene	This study
	with the I229Y substitution	
Y247	JW3973 carrying the *metA* gene	This study
	with the F247Y substitution	
LY	JW3973 carrying the *metA* gene	This study
	with the I124L and I229Y substitutions	
LYD	JW3973 carrying the *metA* gene	This study
	with the I124L, I229Y and N267D substitutions	
WE(P^-^)	WE (∆*clpX-lon)::cat,* ∆*hslVU1172::tet*	This study
L124(P^-^)	L124 (∆*clpX-lon)::cat,* ∆*hslVU1172::tet*	This study
Y229(P^-^)	Y229 (∆*clpX-lon)::cat,* ∆*hslVU1172::tet*	This study
WE∆*dnaK*	WE ∆*dnaK::cat*	This study
L124∆*dnaK*	L124∆*dnaK::cat*	This study
Y229∆*dnaK*	Y229∆*dnaK::cat*	This study
BL21(DE3)	*F- ompT hsdS*_*B*_*(r-*_*B*_*m-*_*B*_*) gal dcm(DE3)*	Novagen (Billerica, USA)
ME7970	∆*hslVU1172::tet*	National Institute
(KY2966)		of Genetics, Japan
Plasmids		
pKD46	*λ* Red (*gam bet exo) ara C rep101*(Ts), Ap^r^	[32]
pMetA	pACYC177 carrying the wild-type *metA*	[11]
	under the natural P_*metA*_ promoter, Ap^r^	
pDnaK	pACYC177 carrying the *dnaK* gene	This study
	under the natural P_*dnaK*_ promoter, Ap^r^	
pPP1	pACYC177 carrying the *clpX-lon* genes	This study
	under the natural P_*clpX*_ promoter, Ap^r^	
pET22b/MetA	Contains the wild-type *metA* gene, Ap^r^	[11]
pET22b/MetAL124	Contains the *metA* gene with I124L substitution, Ap^r^	This study
pET22b/MetAY229	Contains the *metA* gene with I229Y substitution, Ap^r^	This study

*Ap^*r*^ ampicillin resistance, *cat* chloramphenicol resistance gene, *kan* kanamycin resistance gene, *tet* tetracycline resistance gene.

The *E. coli* strains were grown in minimal M9 medium [31] supplemented with glucose (0.2%) or in rich LB medium (Difco Laboratories, Detroit, USA). Antibiotics were used at the following concentrations: ampicillin, 100 μg/ml; chloramphenicol, 20 μg/ml; kanamycin, 25 μg/ml; and tetracycline, 10 μg/ml. L-methionine was added to the medium at a final concentration of 50 μg/ml. The growth of *E. coli* strains in M9 glucose medium at different temperatures was assessed using a TVS126MB automatic growth-measuring incubator (Advantec MFS Inc., Tokyo, Japan). The optical density of the growing cultures was measured at 600 nm every 10 min.

### Site-directed mutagenesis of *metA*

Site-directed mutagenesis was performed using a KOD-Plus-Mutagenesis Kit (Toyobo, Osaka, Japan) according to the manufacturer’s protocol. The plasmid pMetA [11] served as a template, and the primers are shown in Table S6 (Additional file 9). The mutant I229Y was constructed through overlap extension PCR using a QuickChange II-E Site-Directed Mutagenesis Kit (Stratagene, La Jolla, USA) with the primer pair MetY-forward (GCCAGTAAAGATAAGCGCTACGCCTTTGTGACGGG) and MetY-reverse, which is the complement of the forward primer. Changes in the sequence are shown in italic letters.

### Incorporation of the *metA* mutations into the *E. coli* chromosome

The mutated *metA* genes were transferred to the *E. coli* JW3973 (*ΔmetA*) chromosome as previously described [11] using the λ Red recombination system [32].

### Construction of the *∆dnaK::cat* and [(∆*clpX-lon)::cat,* ∆*hslVU1172::tet*] mutants

The structural gene *dnaK* in the WE strain was replaced with the chloramphenicol resistance gene using the λ Red recombination system [32]. A disruption cassette was synthesized through PCR using the forward primer dnaK1 (CAGACTCACAACCACATGATGACCGAATATATAGTGGAGACGTTTAGGTTGGCAGCATCACCCGAC), the reverse primer dnaK2 (CTTCTTCAAATTCAGCGTCGACAACATCGTCATCTTTCGCGTTGTTTGCGTAGCACCAGGCGTTTAAGG), Vent polymerase and the plasmid pACYC184 as a template (homologous sequences are shown in italic letters). Replacement of the *dnaK* gene was confirmed through PCR analysis of the chromosomal DNA of the WE∆*dnaK* strain. A temperature-sensitive phenotype of strain WE∆*dnaK* at 37 and 40°C (data not shown) was rescued with the plasmid pDnak carrying the *dnaK* gene under the endogenous P_*dnaK*_ promoter amplified from the genomic DNA of WE strain using the primers dnaK3 (CGCCTCCTCGAGCATATCGCGAAATTTCTGCGC) and dnaK4 (CCCGTGTCAGTATAATTACCC) and cloned into the XhoI/SmaI restriction sites of the plasmid vector pACYC177. The ∆*dnaK::cat* mutants of strains L124 and Y229 were obtained through transduction with P1*vir* using the WE∆*dnaK* donor strain.

The double mutant ∆*clpX-lon::cat* was constructed after replacing the structural genes in the WE strain with the chloramphenicol resistance gene as previously described [32]. The primers ClpX1-forward (GCATTTGCGTCGTCGTGTGCGGCACAAAGAACAAAGAAGAGGTTTTGACCCGTTGGCAGCATCACCCGAC) and Lon1-reverse (CCTCAATGCGCTTCACAGGATGAATGTCCAGATCGGCAATTACGTTGTCAGGGTAGCACCAGGCGTTTAAGG), Vent polymerase and the plasmid pACYC184 were used to synthesize the chloramphenicol resistance gene flanked by the 51 nucleotides upstream of the *clpX* gene and the 52 nucleotides corresponding with the region 2241–2293 of the *lon* gene (homologous sequences are underlined). The gene *hslVU* in the double mutant ∆*clpX-lon* was replaced through transduction using P1*vir* grown on the ∆*hslVU1172::tet* donor (ME7970), an in-kind gift from the Institute of Genetics, Japan. The resulting strain WE(P^-^) demonstrated temperature sensitive growth at 42°C similar to the previously described triple protease-deficient *E. coli* mutant KY2266 [16]. The normal growth of the WE(P^-^) mutant at 42°C was restored through transformation with the plasmid pPP1 harboring the *clpX-lon* genes under the endogenous P_*clpX*_ promoter amplified from the genomic DNA of WE strain using the primers ClpX4 (CGCCTCCTCGAGCATGCCCGTGAAATTCTG) and Lon4 (GCCATCTAACTTAGCGAGAC) and cloned into the XhoI/SmaI restriction sites of the plasmid vector pACYC177. Replacement of the *clpX, lon, hslVU* genes was confirmed through PCR analysis of the chromosomal DNA of WE(P-) strain. The triple mutants [(∆*clpX-lon)::cat,* ∆*hslVU1172::tet*] of strains L124 and Y229 were obtained through transduction with P1*vir* using the WE(P^-^) donor strain.

### *In vivo* MetA stability analysis

The strains WE, L124 and Y229 were grown in M9 glucose medium at 37°C to the exponential phase (OD_600_ equals 0.3), treated with 200 μg/ml chloramphenicol and divided into two flasks, one of which was shifted to 44°C, while the other flask was maintained at 37°C. The samples were collected before and after chloramphenicol addition every 30 min for 2 h and prepared for Western blotting analysis as previously described [6]. Rabbit anti-MetA antibody (Peptron Inc., Daejeon, Korea) was used as the primary antibody, and horseradish peroxidase-conjugated anti-rabbit IgG antibodies (Pierce, Rockford, USA) were used as the secondary antibody. The immunoblots were developed using a SuperSignal West Pico Chemiluminescent Substrate kit (Pierce, Rockford, USA), scanned with a Fujifilm Image Reader LAS-3000 and analyzed with WCIF ImageJ software.

### Purification of MetA, measurement of enzyme activities and differential scanning calorimetry

The MetA proteins were purified as described previously [11] in the presence of an EDTA-free Halt protease inhibitor cocktail (Pierce, Rockford, USA). To measure the enzyme activities, the decrease in absorbance at 232 nm through the hydrolysis of the thioester bond of succinyl-CoA [3] was monitored using an ND1000 UV/Vis spectrophotometer (Nanodrop Technologies Inc., Wilmington, USA). The enzyme assays were performed in 100 mM K-phosphate buffer (pH 7.5) at 25°C for 30 min in a final volume of 20 μl. The concentrations of the substrates varied from 0.312 mM to 5 mM for L-homoserine and from 0.05 to 0.8 mM for succinyl-CoA. The reactions were initiated after the addition of 0.3 μg of native or mutant MetA.

The thermal stabilities of the MetA proteins were measured calorimetrically over a temperature interval of 15-90°C at a scan rate of 90°C/h with a VP-DSC calorimeter (MicroCal, LLC, Northampton, USA) using 50 μM of protein in a 50 mM K-phosphate buffer (pH 7.5). Three scans were obtained using independent protein preparations.

### *In vitro* MetA aggregation assay

The MetA aggregates were generated after incubating 2 μM of purified protein at 45°C for 30 min, followed by a 40-fold dilution into refolding buffer (50 mM Tris–HCl, pH 7.5, 150 mM KCl, 20 mM MgCl_2_ and 2 mM DTT) [33]. The soluble and insoluble protein fractions were separated through centrifugation at 14,000 g for 30 min. The soluble protein was precipitated with TCA, and the protein pellet was washed twice with ice-cold acetone, dried by speed-vac, dissolved in 20 μl of distilled water and mixed with 20 μl of 2× sample buffer. The samples (10 μl) were loaded onto a 4-15% Criterion™ TGX™ Precast Gel (Bio-Rad, Hercules, USA) and subjected to Western blotting analysis with rabbit anti-MetA antibodies. Densitometry measurements were performed using WCIF ImageJ software.

Purification of soluble and insoluble protein fractions in the heat-stressed cultures The strains WE, L124 and Y229 were grown in M9 glucose medium to exponential phase (approximately OD_600_ = 0.6) at 30°C. Twenty-five milliliters of each culture were shifted to 45°C for 30 min. The remaining 25 ml were used as a control. Aggregated and soluble protein fractions were purified as previously described [34][9] in the presence of EDTA-free Halt protease inhibitor cocktail (Pierce, Rockford, USA). Three micrograms of total protein from the insoluble and soluble fractions were subjected to 12% SDS-PAGE, followed by Western blotting using rabbit anti-MetA antibody. The MetA in the samples was quantified through densitometry using WCIF ImageJ software.

### *In vitro* proteolysis assay

Genes encoding the proteases Lon, ClpP, ClpX, HslU and HslV were cloned into the pET22b expression vector using the primers listed in Table S7 (Additional file 9). Protein was purified using a Ni-NTA Fast Start Kit (Qiagen, Valencia, USA) according to the manufacturer’s protocol. The MetA enzymes and proteases were mixed at the monomer concentrations of 200 pM each in a total of 200 μl of minimal activity buffer (50 mM Tris–HCl, pH 8.0, 10 mM MgCl_2_ and 1 mM DTT) supplemented with an ATP regeneration system (50 mM creatine phosphate and 80 μg/ml creatine kinase (Sigma, St. Louis, USA)) [35]. Degradation was initiated upon the addition of 4 mM ATP at 37°C [35]. The samples were obtained before and after the addition of ATP every hour and analyzed using SDS-PAGE. The band intensities were quantified using WCIF Image J software. The densitometry results were normalized after setting the MetA amount before the ATP addition equal to 100%.

## Competing interests

All authors declare that they have no competing interests.

## Authors’ contributions

EAM and JGP designed and performed all the experiments, collected and interpreted the data and drafted the manuscript. DIK predicted the stabilizing mutation using the computer modeling tools and performed the molecular dynamics analysis of the native and mutated MetA enzymes. All authors read and approved the final manuscript.

## Supplementary Material

Additional file 1: Figure S1 CLUSTAL W (1.83) multiple sequence alignment of the MetA protein sequences from *E. coli* and thermophilic bacteria. Amino acid substitutions in MetA_*E. coli*_ protein are indicated in the boxes. Abbreviations: *Geobacillus - Geobacillus kaustophilus* HTA426 (YP_147640.1|)*; Clostridium - Clostridium thermocellum* ATCC 27405 (YP_001038259.1); *Thermotoga - Thermotoga maritima* ATCC 43589 (NP_228689.1)*; Streptococcus - Streptococcus thermophilus* ATCC 51836 (YP_141582.1)*; Methylococcus - Methylococcus capsulatus* str. Bath (YP_114313.1)*.* Click here for file

Additional file 2: Table S1 Effect of the stabilized MetA mutants on *E. coli* growth at different temperatures. Click here for file

Additional file 3: Figure S2 Effect of multiple mutated MetA enzymes on *E. coli* growth at 45°C. The strains were cultured in M9 glucose medium at 45°C in an automatic growth-measuring incubator. The optical densities of the growing cultures were measured at 600 nm every 10 min. The average of two experiments is presented. Click here for file

Additional file 4: Figure S3 Densitometric analysis of MetAs in the heat-stressed cultures. The *E. coli* strains WE, L124 and Y229 were grown in M9 glucose medium to the exponential phase (approximately OD_600_ = 0.6) at 30°C and subsequently shifted to 45°C for 30 min. Soluble (black columns) and aggregated (gray columns) fractions of MetAs were purified from 25 ml cultures as described in the Methods section. Three micrograms of total protein from the insoluble and soluble fractions were subjected to 12% SDS-PAGE, followed by Western blotting using rabbit anti-MetA antibody. The MetA in the samples was quantified through densitometry using WCIF ImageJ software and normalized to the MetA amount from unstressed cultures, which was equal to 1. The error bars represent the standard deviations of duplicate independent cultures. Abbreviations: Ins, insoluble fraction; Sol, soluble fraction. Click here for file

Additional file 5: Table S2 Effect of the stabilized MetA proteins on growth of the *dnaK* null *E. coli* mutants. **Table S3** Effect of the stabilized MetA proteins on growth of the protease-deficient *E. coli* mutants. Table S4 Effect of the stabilized MetA proteins on growth of the *E. coli* Δ*mukB* mutants. Click here for file

Additional file 6: Figure S4 *In vivo* aggregation of the wild-type and mutated MetAs in heat-stressed cells of the Δ*dnaK* or protease-deficient mutant strains. Aggregates of the wild-type MetA (black columns), mutated I124L (gray columns) and I229Y (dark-gray columns) proteins were purified from the Δ*dnaK* or protease-minus mutants grown in M9 glucose medium at 32°C or 37°C, respectively, to the exponential phase (approximately OD_600_ = 0.6) and transferred to 42°C for 1 h as described in the Methods section. Three micrograms of total protein from the insoluble fractions was subjected to 12% SDS-PAGE, followed by Western blotting using rabbit anti-MetA antibody. The MetAs were quantified through densitometry using WCIF ImageJ software and normalized to the wild-type MetA amount from the WE strain, which was equal to 1. The error bars represent the standard deviations of duplicate independent cultures. Click here for file

Additional file 7: Figure S5 L-methionine eliminates the growth rate difference between the wild-type and stabilized MetAs in Δ*dnaK* or protease-deficient mutants at non-permissive temperatures. The strains were cultured in 25 ml of M9 glucose L-methionine (50 μg/ml) medium in 125 ml Erlenmeyer flasks at 37°C (Δ*dnaK* mutants) or 42°C (protease-minus mutants). The average of two independent experiments is presented. Serial dilutions of cultures growing logarithmically at 30°C (Δ*dnaK* mutants) or 37°C (protease-minus mutants) in M9 glucose medium (OD_600_ of 0.5) were spotted onto M9 glucose L-methionine (50 μg/ml) agar plates. The cells were incubated for 24 h at 37°C (Δ*dnaK* mutants) or 42°C (protease-minus mutants). Click here for file

Additional file 8: Table S5 Free energy and 3D structural analysis of stabilizing single-site mutations of the MetA enzyme. Methods of homology model building and structural analysis of single-site mutated MetA. Click here for file

Additional file 9: Table S6 Primer sequences used for the construction of single-site MetA mutants. **Table S7** Primer sequences employed for the construction of protease expression plasmids. Click here for file
