# Supplementary figures and images for: Stabilized homoserine o-succinyltransferases (MetA) or L-methionine partially recovers the growth defect in Escherichia coli lacking ATP-dependent proteases or the DnaK chaperone
Source: BMC Microbiol. 2013 Jul 30;13:179. doi: 10.1186/1471-2180-13-179 (PMC3735405; doi:10.1186/1471-2180-13-179)

CLUSTAL W (1.83) multiple sequence alignment

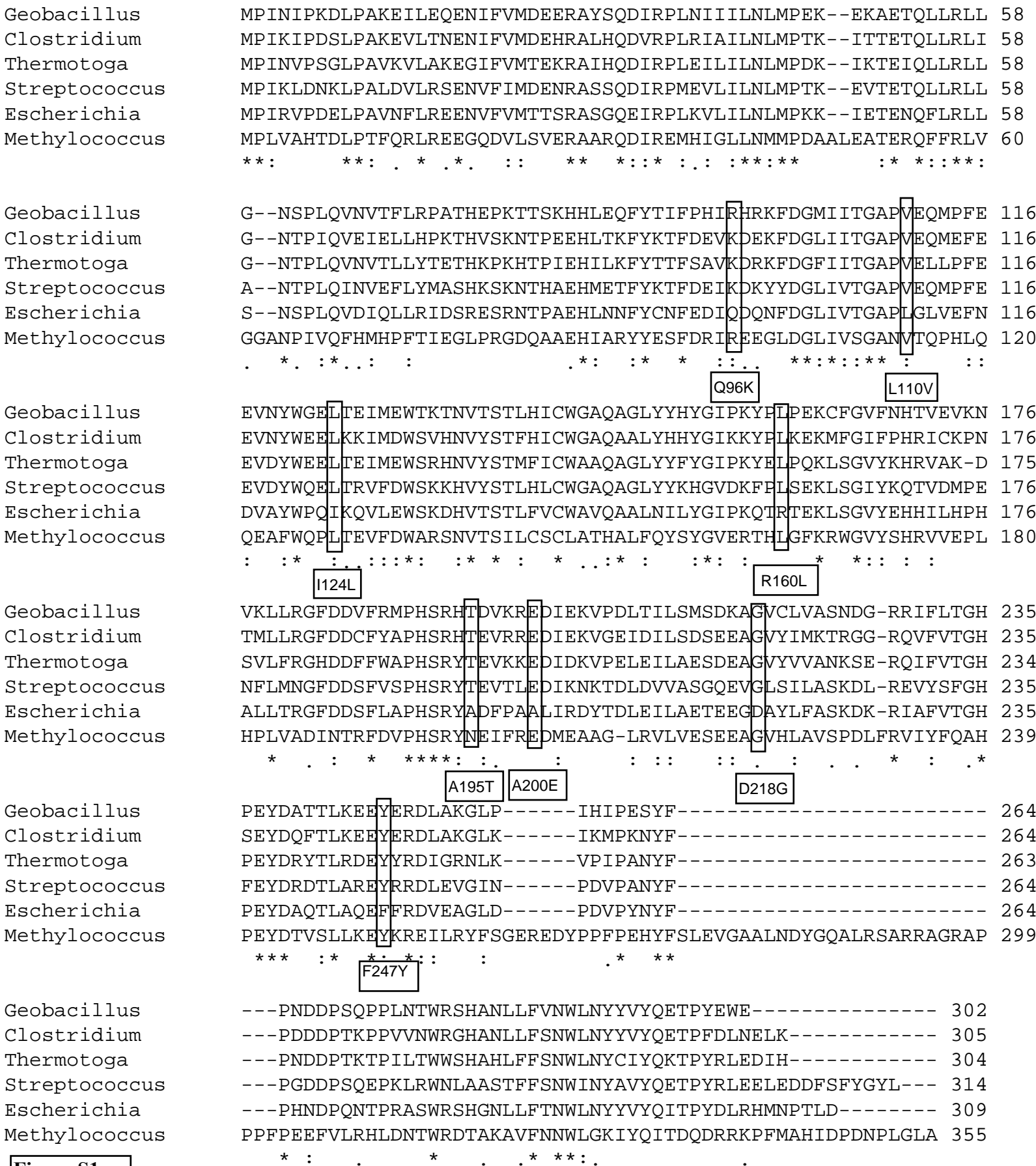

Figure S1.

Supplement: Additional file 1: Figure S1 — CLUSTAL W (1.83) multiple sequence alignment of the MetA protein sequences from E. coli and thermophilic bacteria. Amino acid substitutions in MetAE. coli protein are indicated in the boxes. Abbreviations: Geobacillus - Geobacillus kaustophilus HTA426 (YP_147640.1|); Clostridium - Clostridium thermocellum ATCC 27405 (YP_001038259.1); Thermotoga - Thermotoga maritima ATCC 43589 (NP_228689.1); Streptococcus - Streptococcus thermophilus ATCC 51836 (YP_141582.1); Methylococcus - Methylococcus capsulatus str. Bath (YP_114313.1). [file 1471-2180-13-179-S1.pdf]

## Slide 1
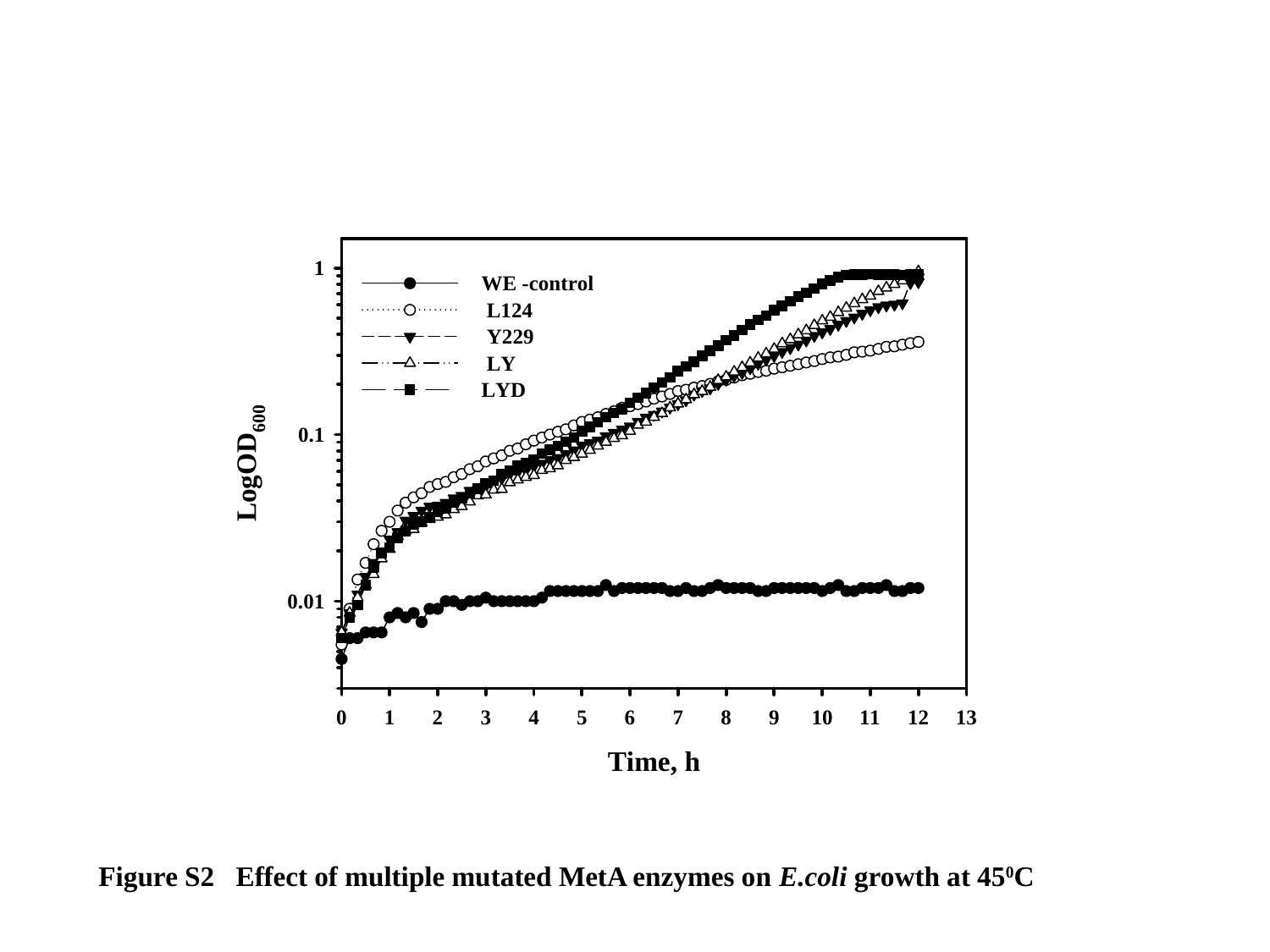

Figure S2 Effect of multiple mutated MetA enzymes on E.coli growth at 450C

Supplement: Additional file 3: Figure S2 — Effect of multiple mutated MetA enzymes on E. coli growth at 45°C. The strains were cultured in M9 glucose medium at 45°C in an automatic growth-measuring incubator. The optical densities of the growing cultures were measured at 600 nm every 10 min. The average of two experiments is presented. [file 1471-2180-13-179-S3.ppt]
