# Supplementary material for: Stabilized homoserine o-succinyltransferases (MetA) or L-methionine partially recovers the growth defect in Escherichia coli lacking ATP-dependent proteases or the DnaK chaperone
Source: BMC Microbiol. 2013 Jul 30;13:179. doi: 10.1186/1471-2180-13-179 (PMC3735405; doi:10.1186/1471-2180-13-179)
Supplement: Additional file 2: Table S1 — Effect of the stabilized MetA mutants on E. coli growth at different temperatures. [file 1471-2180-13-179-S2.doc]

**Table S3 Effect of the stabilized MetA mutants on *E.coli* growth at different temperatures**

**Mutant Specific growth rate μ, h^-1*^, at**

**37^0^C 44^0^C**

Control strain

WE  0.55±0.03 0.16±0.08

Thermostable MetA mutants

K96 0.57±0.014 0.42±0.03▲

L124 0.58±0.014 0.61±0.04▲

Y229 0.58±0.03 0.67±0.025▲

Y247 0.52±0.03 0.51±0.014▲

All the strains were cultured in M9 glucose medium in an automatic growth-measuring incubator, with two repetitions.

^*^The s­­­­­­­­­­pecific growth rate (μ, h^-1^) was calculated by the linear regression analysis of ln(X/X_0_) data with Sigma Plot software, where the initial OD_600_ (X_0_) was 0.15 at the zero time point, and X were the OD_600_ values measured every 10 min in an exponentially growing culture during 1 h.

Symbols: ▼ – decrease of specific growth rate is ≥10% of control strain; ▲ - increase of specific growth rate is ≥10% of control strains.
