# Supplementary material for: Stabilized homoserine o-succinyltransferases (MetA) or L-methionine partially recovers the growth defect in Escherichia coli lacking ATP-dependent proteases or the DnaK chaperone
Source: BMC Microbiol. 2013 Jul 30;13:179. doi: 10.1186/1471-2180-13-179 (PMC3735405; doi:10.1186/1471-2180-13-179)
Supplement: Additional file 4: Figure S3 — Densitometric analysis of MetAs in the heat-stressed cultures. The E. coli strains WE, L124 and Y229 were grown in M9 glucose medium to the exponential phase (approximately OD600 = 0.6) at 30°C and subsequently shifted to 45°C for 30 min. Soluble (black columns) and aggregated (gray columns) fractions of MetAs were purified from 25 ml cultures as described in the Methods section. Three micrograms of total protein from the insoluble and soluble fractions were subjected to 12% SDS-PAGE, followed by Western blotting using rabbit anti-MetA antibody. The MetA in the samples was quantified through densitometry using WCIF ImageJ software and normalized to the MetA amount from unstressed cultures, which was equal to 1. The error bars represent the standard deviations of duplicate independent cultures. Abbreviations: Ins, insoluble fraction; Sol, soluble fraction. [file 1471-2180-13-179-S4.ppt]

## Slide 1
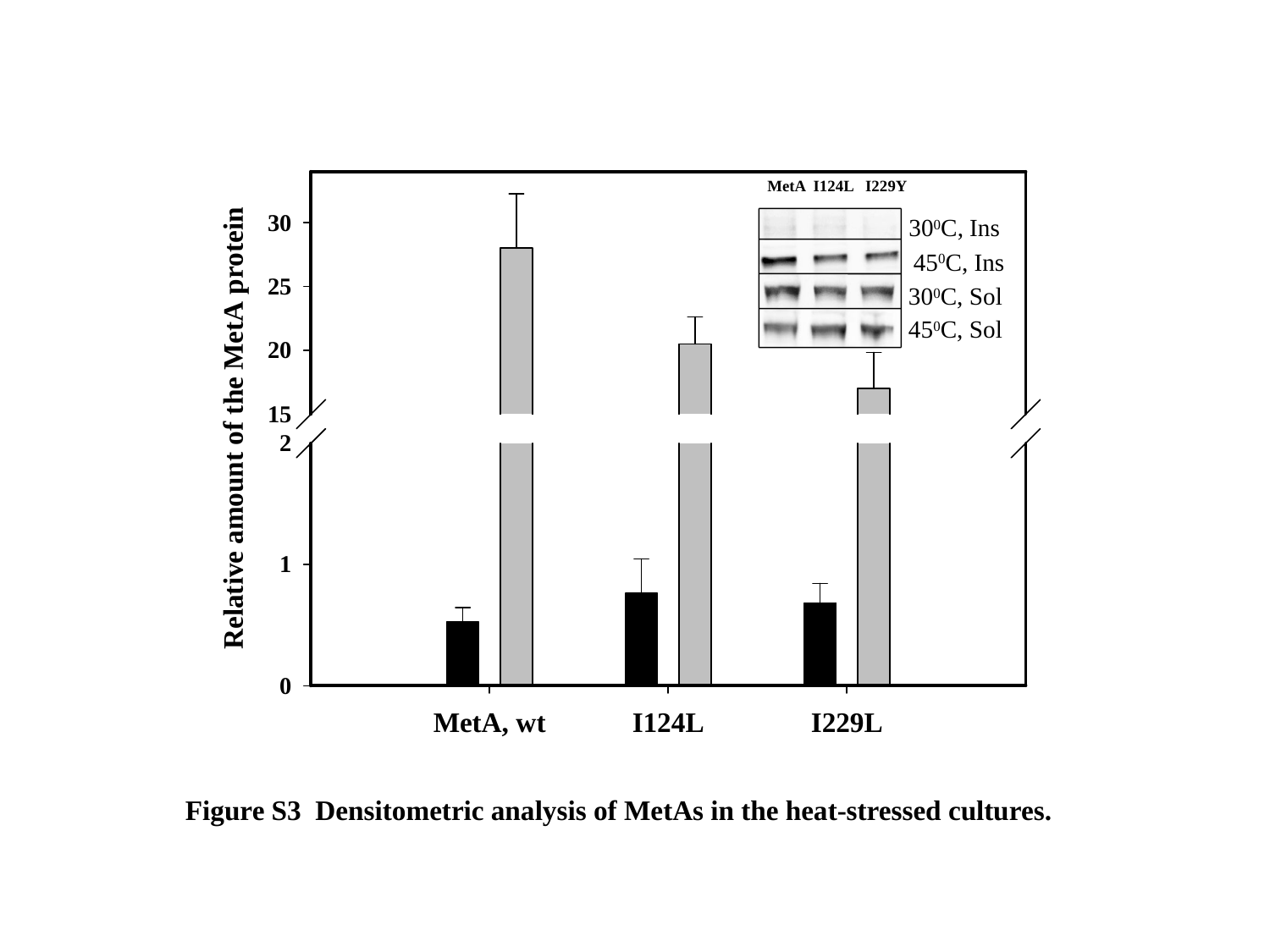

MetA I124L I229Y
300C, Ins
450C, Ins
300C, Sol
450C, Sol
 Figure S3 Densitometric analysis of MetAs in the heat-stressed cultures.
