# Supplementary material for: Stabilized homoserine o-succinyltransferases (MetA) or L-methionine partially recovers the growth defect in Escherichia coli lacking ATP-dependent proteases or the DnaK chaperone
Source: BMC Microbiol. 2013 Jul 30;13:179. doi: 10.1186/1471-2180-13-179 (PMC3735405; doi:10.1186/1471-2180-13-179)
Supplement: Additional file 5: Table S2 — Effect of the stabilized MetA proteins on growth of the dnaK null E. coli mutants. Table S3 Effect of the stabilized MetA proteins on growth of the protease-deficient E. coli mutants. Table S4 Effect of the stabilized MetA proteins on growth of the E. coli ΔmukB mutants. [file 1471-2180-13-179-S5.doc]

**Table S2 Effect of the stabilized MetA proteins on growth of the *dnaK* null *E.coli* mutants.**

**Strain Specific growth rate μ, h^-1^**

**-Methionine +Methionine**

WE 0.6±0.01 0.77±0.01

WEΔ*dnaK* 0.38±0.03 0.69±0.01

L124 0.64±0.01 0.78±0.07

L124Δ*dnaK*  0.48±0.01 0.7±0.01

Y229 0.63±0.01 0.76±0.03

Y229Δ*dnaK*  0.48±0.02 0.65±0.02

The strains were grown in M9 glucose medium at 37^0^C without or with methionine supplementation (50μg/ml). The values shown are the means of duplicate experiments ±SD.

**Table S3 Effect of the stabilized MetA proteins on growth of the protease-deficient *E.coli* mutants.**

**Strain Specific growth rate μ, h^-1^**

**-Methionine +Methionine**

WE 0.54±0.01 0.78±0.03

WE(P-) 0.096±0.01 0.58±0.02

L124 0.64±0.03 0.79±0.04

L124(P-) 0.23±0.03 0.6±0.01

Y229 0.65±0.01 0.8±0.03

Y229(P-) 0.25±0.01 0.59±0.03

The strains were grown in M9 glucose medium at 42^0^C without or with methionine supplementation (50μg/ml). The values shown are the means of duplicate experiments ±SD.

**Table S4 Effect of the stabilized MetA proteins on growth of the *E.coli* Δ*mukB* mutants**

**Strain Specific growth rate μ, h^-1^**

WE 0.53±0.04

WEΔ*mukB*  0.42±0.02

Y229 0.61±0.02

Y229Δ*mukB*  0.52±0.01

L124 0.6±0.04

L124Δ*mukB* 0.49±0.03

WE+methionine 0.7±0.07

WEΔ*mukB+*methionine 0.6±0.02

Overnight cultures grown in M9 glucose medium at 30^0^C were inoculated into fresh M9 glucose medium and cultivated in flasks at 41^0^C. The values shown are the means of duplicate experiments ±SD.
