# Supplementary material for: Stabilized homoserine o-succinyltransferases (MetA) or L-methionine partially recovers the growth defect in Escherichia coli lacking ATP-dependent proteases or the DnaK chaperone
Source: BMC Microbiol. 2013 Jul 30;13:179. doi: 10.1186/1471-2180-13-179 (PMC3735405; doi:10.1186/1471-2180-13-179)
Supplement: Additional file 6: Figure S4 — In vivo aggregation of the wild-type and mutated MetAs in heat-stressed cells of the ΔdnaK or protease-deficient mutant strains. Aggregates of the wild-type MetA (black columns), mutated I124L (gray columns) and I229Y (dark-gray columns) proteins were purified from the ΔdnaK or protease-minus mutants grown in M9 glucose medium at 32°C or 37°C, respectively, to the exponential phase (approximately OD600 = 0.6) and transferred to 42°C for 1 h as described in the Methods section. Three micrograms of total protein from the insoluble fractions was subjected to 12% SDS-PAGE, followed by Western blotting using rabbit anti-MetA antibody. The MetAs were quantified through densitometry using WCIF ImageJ software and normalized to the wild-type MetA amount from the WE strain, which was equal to 1. The error bars represent the standard deviations of duplicate independent cultures. [file 1471-2180-13-179-S6.ppt]

## Slide 1
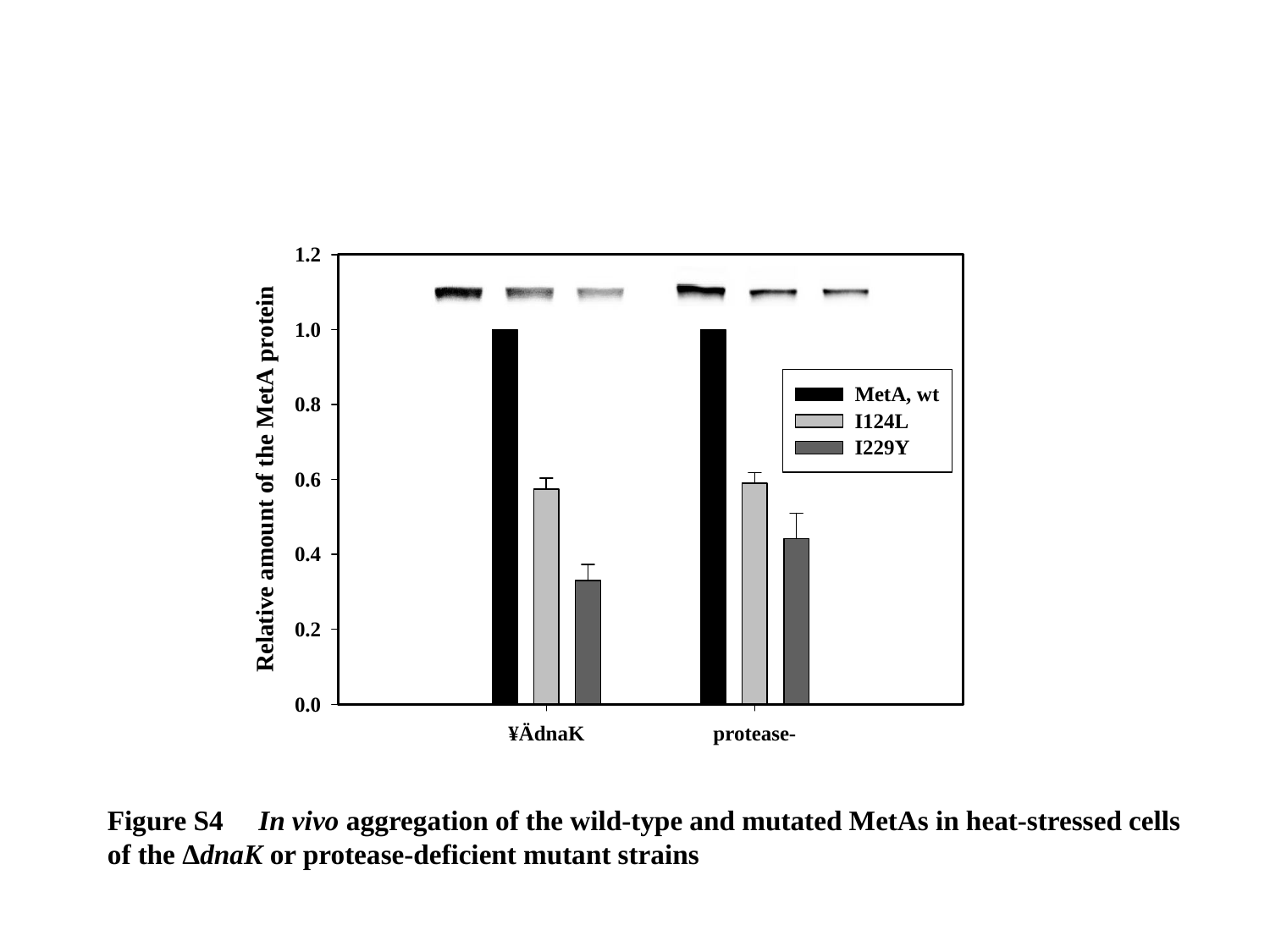

Figure S4 In vivo aggregation of the wild-type and mutated MetAs in heat-stressed cells of the ΔdnaK or protease-deficient mutant strains
