# Supplementary material for: Stabilized homoserine o-succinyltransferases (MetA) or L-methionine partially recovers the growth defect in Escherichia coli lacking ATP-dependent proteases or the DnaK chaperone
Source: BMC Microbiol. 2013 Jul 30;13:179. doi: 10.1186/1471-2180-13-179 (PMC3735405; doi:10.1186/1471-2180-13-179)
Supplement: Additional file 7: Figure S5 — L-methionine eliminates the growth rate difference between the wild-type and stabilized MetAs in ΔdnaK or protease-deficient mutants at non-permissive temperatures. The strains were cultured in 25 ml of M9 glucose L-methionine (50 μg/ml) medium in 125 ml Erlenmeyer flasks at 37°C (ΔdnaK mutants) or 42°C (protease-minus mutants). The average of two independent experiments is presented. Serial dilutions of cultures growing logarithmically at 30°C (ΔdnaK mutants) or 37°C (protease-minus mutants) in M9 glucose medium (OD600 of 0.5) were spotted onto M9 glucose L-methionine (50 μg/ml) agar plates. The cells were incubated for 24 h at 37°C (ΔdnaK mutants) or 42°C (protease-minus mutants). [file 1471-2180-13-179-S7.ppt]

## Slide 1
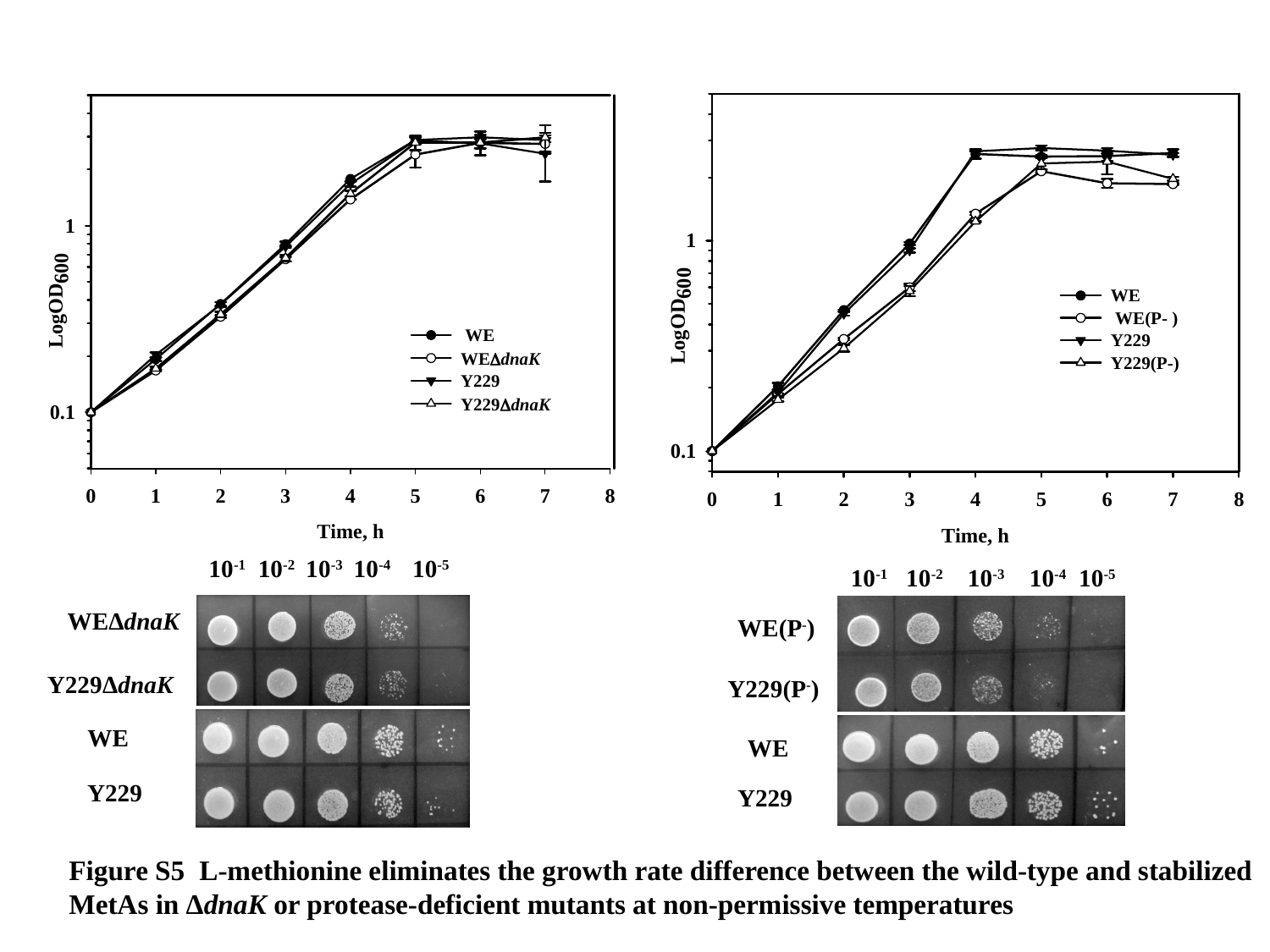

10-1 10-2 10-3 10-4 10-5
 10-1 10-2 10-3 10-4 10-5
WEΔdnaK
WE(P-)
Y229ΔdnaK
Y229(P-)
WE
WE
Y229
Y229
Figure S5 L-methionine eliminates the growth rate difference between the wild-type and stabilized
MetAs in ΔdnaK or protease-deficient mutants at non-permissive temperatures
