# Supplementary material for: Stabilized homoserine o-succinyltransferases (MetA) or L-methionine partially recovers the growth defect in Escherichia coli lacking ATP-dependent proteases or the DnaK chaperone
Source: BMC Microbiol. 2013 Jul 30;13:179. doi: 10.1186/1471-2180-13-179 (PMC3735405; doi:10.1186/1471-2180-13-179)
Supplement: Additional file 8: Table S5 — Free energy and 3D structural analysis of stabilizing single-site mutations of the MetA enzyme. Methods of homology model building and structural analysis of single-site mutated MetA. [file 1471-2180-13-179-S8.doc]

**Table S5 Free energy and 3D structural analysis of stabilizing single-site mutations of the MetA enzyme­­­­­­­­­­­­­­­**

**Mutations**

**Q96K I124L I229Y F247Y**

^a^SS Other Helix Sheet Helix

^b^SAS_WT_ (Å^2^) 53.75% 1.08% 13.51% 13.95%

SAS_Mut_(Å^2^) 56.88% 0.0% 5.15% 21.06%

Results Exposed Buried Buried Buried

^c^ΔΔG(kcal/mol) -1.37 -1.63 -2.23 -1.53

^d^V_WT_(Å^3^) 105.374 ± 1.35 106.908 ± 0.81 149.034 ± 2.25 102.424± 2.07

V_Mut_(Å^3^) 100.311± 1.17 99.963 ± 0.18 95.613± 2.43 72.297 ± 2.52

Surrounding Phe92 Leu41 Leu128 Leu243

residues Glu93 Leu43 Glu129 Ala244

Asp94 Tyr120 Ser131 Gln245

Ile95 Trp121 Lys132 Glu246

Gln98 Pro122 Ser137 Phe248

Trp130 Gln123 Thr138 Arg249

His134 Lys125 Leu139 Asp250

Val127 Ala223 Val251

Leu128 Ser224 Pro257

Lys227 Asp258

Arg228 Val259

Ala230 Pro269

Phe231

^a^SS, secondary structure.

^b^SAS_WT_ (Å^2^) and SAS_Mut_ (Å^2^), the solvent accessible surface area of the wild-type MetA and its mutants, respectively.

^c^ΔΔG (kcal/mol), free energies of each mutants for MetA were calculated by thermodynamic perturbation methods.

^d^V_WT_(Å^3^) and V_Mut_ (Å^3^), the cavity volume of the wild-type MetA and its mutants,

respectively.

**Homology model building and structural analysis of single-site mutated MetA**

The MODELLER program [1] was used to generate 50 models of the MetA protein. The quality control tool of the “What If” program [2] was employed to evaluate these models and select the best. The structures were geometry minimized using the CHARMM [3] molecular mechanics force field to within an Root Mean Square (RMS) gradient of 0.5 kcal mol^-1^Å^-1^, formed by adding a sphere of water molecules containing a layer of 10 Å. Throughout, all systems were also surrounded by a distance-dependent dielectric model. To obtain relaxed geometries, short molecular dynamics (MD) were simulated, followed by a final energy minimization step. MD simulations of the MetA protein and its mutants were performed using a canonical ensemble, NVT, with an initial temperature 150 K; heating for 50 ps; simulation temperature 300 K; duration 500 ps; time-step 1 fs; temperature response 1 ps; pressure response 0.5 ps, and constraint tolerance 1 × 10^-9^ ps.

The conformations of the mutant MetAs (Q96K, I124L, I229Y, F247Y) were constructed from the minimized wild-type structure by substituting each amino acid for the normally occurring amino acid at the respective position in the wild-type structure. In each case, the backbone dihedral angles for the substituted residue were the same as for the corre­sponding amino acid in the wild-type protein. The start­ing side-chain conformation for the substituted residue was at the lowest energy level for the given backbone conformation, as determined by the rotamer library [4]. This structure was subjected to nested molecular dynamics and energy minimizations using CHARMM [3] potential functions. Dynamics were run for a total of 2 ns, and the total conformational energies for each mutant structure con­verged to a minimum value. The last 50 isoenergetic structures on the trajectory were employed in computing the average structure and coordinate fluctuations. The average structure for each mutant form was superim­posed on that of the wild-type protein, such that the RMS deviation of the coordinates of the backbone atoms of one structure from the other was at a minimum. The average RMS deviation between the mutant and the wild-type proteins was determined for each amino acid residue. Similarly, the average structure for each mutant form was superimposed on every other mutant form and the RMS deviations determined.

Free energies were calculated by applying the free energy perturbation (FEP) method [5-7]. To calculate free energy differences between the wild-type and mutated MetAs, a thermodynamic cycle was carried out of free energy simulations for alchemical transformation in which one amino acid was transformed into another. These were realized by running the PERT module in CHARMM [3] that used the single topology approach, in which every atom in the initial state has a counterpart in the final state. FEP simulations were carried out by defining a series of non-physical intermediate points between the initial and final state (reference and mutated MetA) and calculating the sum of free energy change computed for each step, using double-wide sampling (unless different equilibration or production time was specified). Several independent free energy simulations were performed for each type of mutation that differed in initial velocities or starting conformation, taken from different snapshots of the same trajectory or independent trajectories from previously performed dynamics of the MetA protein. Several models of the MetA were examined in the calculation of free energy changes due to mutation in the wild-type enzyme. At least one simulation in the backward direction was performed for each mutation. FEP simulations were run in the NPT ensemble at 1 atm and 300 K, using extended system constant pressure [8], temperature algorithm, time-step 1 fs, and SHAKE algorithm applied only to the hydrogens of water. The temperature was controlled by the Hoover method [9].

**REFERENCES**

1. Eswar N, Marti-Renom MA, Webb B, Madhusudhan MS, Eramian D, Shen M, Pieper U, Sali A: **Comparative protein structure modeling with MODELLER.** In *Current Protocols in Bioinformatics.* New Jersey: John Wiley & Sons; 2006, Supplement 15, 5.6.1-5.6.30.

2.Vriend G: **WHAT IF: A molecular modeling and drug design program.** *J Mol Graph* 1990, **9**: 52-56.

3. MacKerell JrDA, Brooks B, Brooks II CL, Nilsson L, Roux B, Karplus M: **CHARMM: the energy function and its parameterization with an overview of the program.** In *Encyclopedia of Computational Chemistry, Volume 2.* Edited by von Ragué Schleyer P, Allinger NL, Clark T, Gasteiger J, Kollman PA, Schaefer III HF. Chichester, UK: John Wiley & Sons; 1998, 271-277.

4.Dunbrack RLJr: **Rotamer libraries in the 21st century.** *Curr Opin Struct Biol* 2002, **12**: 431-440.

5.Kollman P: **Free energy calculations: applications to chemical and biochemical phenomena.** Chem Rev 1993, **93**: 2395-2417.

6.Mark AE: **Free energy perturbation calculation.** In *Encyclopedia of Computational Chemistry, Volume 2.* Edited by von Ragué Schleyer P, Allinger NL, Clark T, Gasteiger J, Kollman PA, Schaefer III HF. Chichester, UK: John Wiley & Sons; 1998, 1070-1083.

7.Pearlman DA, Govinda R: **Free energy calculations: methods and applications**. In *Encyclopedia of Computational Chemistry, Volume 2.* Edited by von Ragué Schleyer P, Allinger NL, Clark T, Gasteiger J, Kollman PA, Schaefer III HF. Chichester, UK: John Wiley & Sons; 1998, 1036-1061.

8.Feller SE, Zhang Y, Pastor RW, Brooks BR: **Constant pressure molecular dynamics simulation: the Langevin piston method.** *J Chem Phys* 1995, **103**: 4613-4621.

9.Hoover WG: **Canonical dynamics: equilibrium phase-space distributions.** *Phys Rev A* 1985, **31**: 1695-1697.
