# Supplementary material for: Stabilized homoserine o-succinyltransferases (MetA) or L-methionine partially recovers the growth defect in Escherichia coli lacking ATP-dependent proteases or the DnaK chaperone
Source: BMC Microbiol. 2013 Jul 30;13:179. doi: 10.1186/1471-2180-13-179 (PMC3735405; doi:10.1186/1471-2180-13-179)
Supplement: Additional file 9: Table S6 — Primer sequences used for the construction of single-site MetA mutants. Table S7 Primer sequences employed for the construction of protease expression plasmids. [file 1471-2180-13-179-S9.doc]

**Table S6 Primer sequences used for the construction of single-site MetA mutants**

**Primer Mutation Sequence**

K3-forward Q96K **A**AGGATCAGAACTTTGACGGTTTG

K3–reverse Q96K AATATCTTCAAAGTTACAGTAGAAG

V3-forward L110V G**G**TGGGCCTGGTGGAGTTTAATG

V3-reverse L110V GGCGCACCAGTTACAATCAAACC

L2-forward I124L CAG**C**TCAAACAGGTGCTGGAGTG

L2-reverse I124L CGGCCAGTAAGCGACATCATTAAAC

L1-forward R160L CTC**T**CACCGAAAAACTCTCTGGC

L1–reverse R160L TTTGCTTAGGAATGCCGTAGAGG

T4-forward A195T CTAT**A**CTGACTTTCCGGCAGCGTTG

T4-reverse A195T CGCGAATGCGGTGCCAGGAATGAATC

E1-forward A200E CAG**A**GTTGATTCGTGATTACACCG

E1–reverse A200E CCGGAAAGTCAGCATAGCGCGAATG

G1–forward D218G G**G**TGCATATCTGTTTGCCAGTAAAG

G1–reverse D218G CCCTTCTTCCGTCTCTGCCAGAATTTC

Y2-forward F247Y GAAT**A**TTTCCGCGATGTGGAAGCC

Y2-reverse F247Y CTGCGCCAGCGTTTGCGCATCATATTC

The mutation sites are underlined in bold.

**Table S7 Primer sequences employed for construction of proteases’ expression plasmids**

­­­­­­­­­­­­­­

**Primer Sequence Restriction enzymes**

Lon2 CGCCTCATTAATATGAATCCTGAGCGTTCTGAACGC *Ase*I

Lon3 CGCCTCGAATTCGGTTTTGCAGTCACAACCTGCATACC *Eco*RI

ClpP1 CGCCTCATTAATATGTCATACAGCGGCGAACG *Ase*I

ClpP2 CGCCTCAAGCTTATTACGATGGGTCAGAATCGAATC *Hind*III

ClpX2 CGCCTCCATATGACAGATAAACGCAAAGATGGC *Nde*I

ClpX3 CGCCTCAAGCTTTTCACCAGATGCCTGTTGCGCTTC  *Hind*III

HslV1 CGCCTCCATATGACAACTATAGTAAGCGTACG  *Nde*I

HslV2 CGCCTCAAGCTTCGCTTTGTAGCTTAATTCTTCGATG *Hind*III

HslU1 CGCCTCCATATGTCTGAAATGACCCCACGC *Nde*I

HslU2 CGCCTCAAGCTTTAGGATAAAACGGCTCAGATCTTC  *Hind*III

The restriction sites are underlined.
